# Computational Studies of Difference in Binding Modes of Peptide and Non-Peptide Inhibitors to MDM2/MDMX Based on Molecular Dynamics Simulations

**DOI:** 10.3390/ijms13022176

**Published:** 2012-02-17

**Authors:** Jianzhong Chen, Dinglin Zhang, Yuxin Zhang, Guohui Li

**Affiliations:** 1 Laboratory of Molecular Modeling and Design, State Kay Laboratory of Molecular Reaction Dynamics, Dalian Institute of Chemical Physics, Chinese Academy of Science, Dalian 116011, China; E-Mails: chenjianzhong1970@163.com (J.C.); dlzhang@dicp.ac.cn (D.Z.); zyx19840227@yahoo.cn (Y.Z.); 2 Department of Mathematics and Physics, Shandong Jiaotong University, Jinan 250031, China

**Keywords:** p53-MDM2/MDMX interaction, molecular dynamics simulation, binding free energy, alanine scanning

## Abstract

Inhibition of p53-MDM2/MDMX interaction is considered to be a promising strategy for anticancer drug design to activate wild-type p53 in tumors. We carry out molecular dynamics (MD) simulations to study the binding mechanisms of peptide and non-peptide inhibitors to MDM2/MDMX. The rank of binding free energies calculated by molecular mechanics generalized Born surface area (MM-GBSA) method agrees with one of the experimental values. The results suggest that van der Waals energy drives two kinds of inhibitors to MDM2/MDMX. We also find that the peptide inhibitors can produce more interaction contacts with MDM2/MDMX than the non-peptide inhibitors. Binding mode predictions based on the inhibitor-residue interactions show that the π–π, CH–π and CH–CH interactions dominated by shape complimentarity, govern the binding of the inhibitors in the hydrophobic cleft of MDM2/MDMX. Our studies confirm the residue Tyr99 in MDMX can generate a steric clash with the inhibitors due to energy and structure. This finding may theoretically provide help to develop potent dual-specific or MDMX inhibitors.

## 1. Introduction

The tumor suppressor protein p53, “the guardian of the genome,” plays a key role in maintaining the integrity of the genome by inducing cell cycle arrest and apoptosis in response to stress [1]. It can protect higher organisms from cancer by activating its original function [2]. However, the oncoproteins MDM2 and MDMX negatively regulate the activity of the tumor suppressor p53 by binding to the *N*-terminal transactivation domain of p53 [3–6]. In fact, the overexpressions of MDM2 and MDMX contribute to the loss of p53 activation and tumor survival in tumors [7,8]. Almost 50% of all human cancers are due to invalidation of the p53 function caused by deletions or mutations in the DNA-binding domain of p53 [9]. Thus, the p53-MDM2/MDMX interaction becomes an attractive molecular target for cancer therapy.

**Figure 1:**
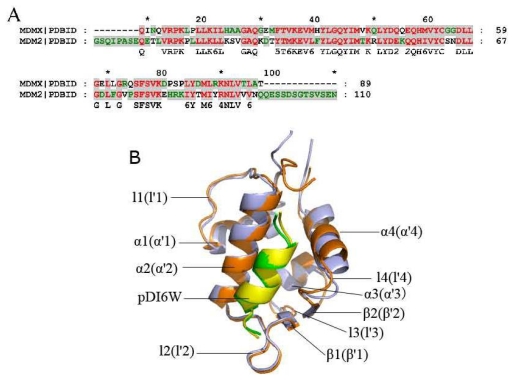
Sequence and structure of the binding domain of MDM2/MDMX to p53. (**A**) The sequence alignment of MDM2 with MDMX; (**B**) Stereoview of superimposed structures of pDI6W-MDM2 (green/orange) and pDI6W-MDMX (yellow/light blue) in a cartoon diagram. The sign “′” represents the second structure belonging to MDMX. In Figure 1B, α β and l represent α-helix, β-sheet and loop, respectively.

Recent studies show that MDMX is not only highly homologous (55%) to MDM2 (Figure 1A), but also shares a common structural sequence of a ααβαβα topology in overall structures of human MDM2/MDMX (Figure 1B) [10–12]. Superposition of MDM2 and MDMX displays a large structural difference in α4 (α′4) helix, stemming from a change of His96 of MDM2 to Pro95 of MDMX [13].

Earlier biochemical studies using p53 peptide have proven that MDM2 binds to p53 with 10-fold higher affinity than MDMX [14]. Previous insights into the inhibition of the p53-MDM2/MDMX interaction also reveal that the most of the peptide and non-peptide inhibitors, such as Nutlins and MI-219, provide strong anti-tumor potential of MDM2 inhibitors, but they do not efficiently inhibit the interaction of MDMX with p53 [6,14–24]. Lately, a 15-residue p53 peptide determined by Popowicz *et al.* shows similar affinity to MDM2/MDMX [25]. This provides a possibility of developing dual inhibitors of the p53-MDM2/MDMX interaction. Furthermore, this result has been supported by the studies of several other groups [16,26–32].

**Figure 2:**
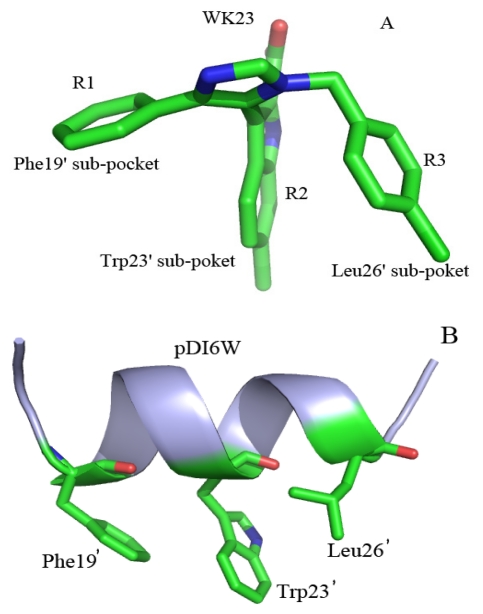
Structures of inhibitors. (**A**) Non-peptide inhibitor WK23 is shown in sticks and green; (**B**) peptide inhibitor pDI6W is shown in cartoon and light blue, and three residues are shown in stick and green.

Understanding the binding mechanisms of the peptide and non-peptide inhibitors to MDM2/MDMX at an atomic level may facilitate the development of potent dual inhibitors inhibiting the p53-MDM2/MDMX interaction and provide valuable information about the structure-affinity relationships of the p53-MDM2/MDMX complexes. A few computational studies have been performed for this purpose [26,33,34]. In this work, we selected a peptide inhibitor pDI6W and a non-peptide inhibitor WK23 to probe the difference in the binding mechanisms of two kinds of inhibitors to MDM2/MDMX. WK23 is an inhibitor based on four aromatic groups studied by Popowicz G.M. *et al.* and able to efficiently fill the binding pockets of MDM2/MDMX, its median inhibitory concentration (IC_50_) values to MDM2/MDMX are 1.17 and 36 μM, respectively [6]. pDI6W is a 12-residue peptide inhibitor (LTFEHWWAQLTS) designed by Phan J. *et al.* with IC_50_ values of 36 and 250 nM to MDM2/MDMX, respectively [31]. Both of the two inhibitors have big differences in binding free energies to MDM2 and MDMX [6,31]. Thus it is significant to explore the reason for this difference for the design of dual inhibitors. Figure 2 depicts the structures of two inhibitors and points out the parts imitating three residues of p53: Phe19′, Trp23′, and Leu26′, inserted into the hydrophobic groove in MDM2/MDMX.

Binding free energy calculations have been proven to be powerful and valuable tools for understanding the binding mechanisms of inhibitors to proteins. To date, several effective methods have been proposed to calculate the binding free energies of protein inhibitors: free energy perturbation (FEP) [35], thermodynamic integration (TI) [36,37] and MM-PB(GB)SA *etc.* [21,38–41]. Although FEP and TI should give more accurate binding free energies, they are restricted to closely related chemical structures of inhibitors. Furthermore, MM-PB(GB)SA method has been used successfully in explaining protein-protein and protein-inhibitor interactions [28,42–47]. In this method, polar solvation free energy calculated by the Possion-Boltzmann (PB) equation leads MM-PBSA calculations, while obtained by the generalized Born equation is the so-called MM-GBSA calculations [48–50]. Thus, in this work, the MM-GBSA method combined MD simulation was applied to calculate the binding free energies of two inhibitors to MDM2/MDMX. By the calculations of the binding free energy, the inhibitor-residue interaction and alanine scanning, we expect that the following three aims can be achieved: (1) to understand the difference in the binding modes of two different kinds of inhibitors; (2) to illuminate the main force to drive the bindings of inhibitors in the hydrophobic cleft of MDM2/MDMX; (3) to explore the cause of a big difference in the binding free energy of the same inhibitor to MDM2/MDMX with high homology and similar structure. We also expect that this study can provide important hints for the design of the potent dual inhibitor inhibiting the interaction of p53 with MDM2/MDMX.

## 2. Results and Discussion

### 2.1. System Stability During MD Simulations

**Figure 3:**
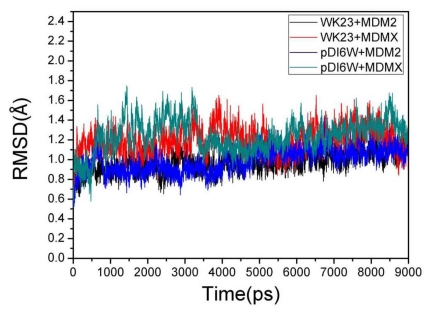
Root-mean-square deviations (RMSD) of backbone atoms relative to their initial minimized structures as function of time.

To evaluate the reliable stability of MD trajectories, RMSD of backbone atoms relative to the initial minimized structure through the phase of the simulation was plotted in Figure 3. One can see that four complexes have reached the equilibrium about after 4.5 ns of the simulation phase. According to Figure 3, the RMSD values of WK23-MDM2, pDI6W-MDM2, WK23-MDMX and pDI6W-MDMX complexes are 1.07, 1.08, 1.19 and 1.27 Å, respectively, with a deviation of lower than 0.65 Å. This result shows that the trajectories of MD simulations for four complexes after the equilibrium are reliable for post analyses. It was observed from Figure 3 that the RMSD values of two complexes involving MDM2 are lower than MDMX.

### 2.2. Superimposition Analyses

**Figure 4:**
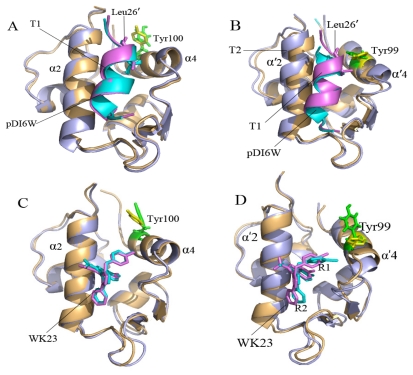
Crystal structures were superimposed on the average structure from the last 500 ps of molecular dynamics simulation at an interval of 10 ps, (**A**) for the pDI6W-MDM2 complex; (**B**) for the pDI6W-MDM2 one; (**C**) for the WK23-MDM2 one; and (**D**) for the WK23-MDMX one. In the average structure, protein, inhibitor and Tyr100 (Tyr99) are shown in light orange, cyan and green, respectively. In the crystal structure, the protein, inhibitor and Tyr100 (Tyr99) are displayed in light blue, violet and yellow. The residue Leu26′ is from pDI6W and second structure labeled by α′ belongs to MDMX. T1 represents the residues 49–51 in MDMX and T2 indicates the residues 24 and 25 in pDI6W.

To acquire an atomic view of the difference between the MD-simulated structures and crystal structures, the structures of the MD-simulated complexes from the last 500 ps of MD simulations at an interval of 10 ps were superimposed with the crystal structures (plotted in Figure 4). As shown in Figure 4, the residue Tyr100 in the helix α4 of MDM2 moves obviously, but except for the slight shift of the position T1 in the inhibitor pDI6W, the MD-simulated structures of MDM2 agree well with the crystal structures (Figure 4A,C). In the case of the pDI6W-MDMX complex, the residue Tyr99 in the helix α′4 of MDMX and pDI6W have slight shifts from the crystal structure, though the helix α′4 and the end T2 of α′2 in MDMX obviously depart from the crystal structure. Although the residue Tyr99, the ring R1 and R2 of the inhibitor WK23 and the helix α′4 highly deviate in their crystal structures in the case of the WK23-MDMX complex, the remainder of MDMX takes the same orientation as in the crystal structure. To sum up, the superimposition analyses suggest that the MD-simulated structures of MDM2 have smaller deviation from the crystal structure than MDMX, which agrees basically with the previous RMSD analyses.

### 2.3. Calculations of Binding Free Energies

To further evaluate the difference in the binding modes of the inhibitors to MDM2/MDMX and obtain detailed insights into the contribution of each component to the inhibitor-protein binding, the binding affinities of the inhibitors to MDM2/MDMX were estimated by performing MM-GBSA calculations based on a single-trajectory MD simulation. Because the radius parameter of chlorine atoms is missing for MM-GBSA module in Amber 10, we add the radii of 1.75 Å for chlorine to pbsa program in Amber [51]. The calculated results and experimental data (Δ*G*_exp_) were summarized in Table 1. The predicted binding free energies of pDI6W-MDM2, pDI6W-MDMX, WK23-MDM2 and WK23-MDMX complex are −21.93, −19.74, −16.81 and −14.89 kcal·mol^−1^, respectively. Furthermore, it is encouraging that the ranking of the experimental binding free energies are consistent with our predictions, which suggests the MD-simulation models and computational protocol tested in this study is reliable.

As shown in Table 1, the major favorable contributors to the inhibitor binding are van der Waals energies (Δ*G*_vdw_). Non-polar solvation energies (Δ*G*_nopol_), which correspond to the burial of SASA upon binding, also provide important contributions to binding. However, the contributions of the entropy changes to the free energies (−*T*Δ*S*) impair the bindings of two inhibitors to MDM2/MDMX. Although the electrostatic terms (Δ*G*_ele_) favored inhibitor binding, these favorable contributions were completely screened by the unfavorable stronger polar solvation energies (Δ*G*_pol_). It is noted that the electrostatic interaction of pDI6W with MDM2/MDMX is two times stronger than van der Waals energies, while the electrostatic terms of the interaction between WK23 and MDM2/MDMX is much weaker than van der Waals energy. Thus, it is concluded van der Walls energy dominates the bindings of the inhibitors in the hydrophobic cleft of two oncoproteins. This result agrees with the previous studies of several groups [6,26,34].

**Figure 5:**
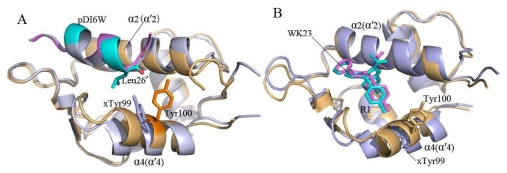
Stereoview of superimposed structures of inhibitor-MDM2 complex and inhibitor-MDMX complex. MDM2/MDMX and pDI6W are displayed in a cartoon mode, MDM2 is shown in light orange and MDMX in light blue, Leu26′, Tyr99 of MDMX and Tyr100 of MDM2 are shown in stick. (**A**) pDI6W-MDM2/MDMX complex; (**B**) WK23-MDM2/MDMX complex. The character “x” before the residue represents this residue belongs to MDMX.

Two interesting phenomena are observed from Table 1. (1) Compared with the interactions of the non-peptide inhibitor WK23 with MDM2/MDMX, the electrostatic interaction and van der Waals term of pDI6W with MDM2 and MDMX are much stronger than WK23. This result shows that the peptide inhibitor pDI6W can produce more interaction contacts with MDM2/MDMX than the non-peptide inhibitor WK23; (2) The van der Waals interactions of pDI6W and WK23 with MDMX are 2.17 and 3.79 kcal·mol^−1^, weaker than one of pDI6W and WK23 with MDM2, respectively. Despite a high homology and similar structure of MDM2/MDMX (Figure 1B), the bindings of the same inhibitor result in an obvious decrease in van der Waals energy. To explain this phenomenon, we analyzed the conformations of all residues in the helix α4/α′4 from MDM2/MDMX and observed that the residue Tyr99 in MDMX takes very different side chain orientation from the residue Tyr100 in MDM2 (Figure 5). For MDM2, the side chain of Tyr100 points outward and accommodates the bindings of Leu26′ and the ring R1 of WK23. However, for MDMX, the side chain of Tyr99 orients toward Leu26′ and the ring R1 of WK23, which shows that Tyr99 prevents the inhibitor from moving into the deep cleft between α′2 and α′4 of MDMX, and generates less inhibitor-MDMX contacts, this leads to a decrease in van der Waals energy. The above analyses basically agree with the previous studies [26,31,32].

### 2.4. Binding Mode Predictions of Inhibitors to MDM2/MDMX

**Figure 6:**
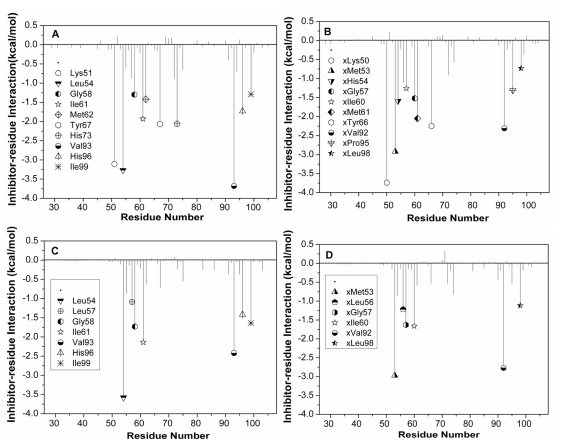
Inhibitor-residue interaction spectrum of (**A**) pDI6W-MDM2 complex; (**B**) pDI6W-MDMX complex; (**C**) WK23-MDM2 complex; and (**D**) WK23-MDMX complex based on MM-GBSA method. The residues with interaction energy of larger than 1 kcal·mol^−1^ are labeled. The character “x” before the residue represents this residue belongs to MDMX.

**Figure 7:**
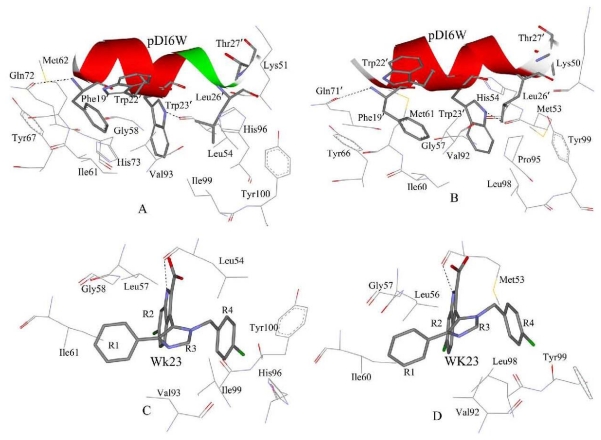
Geometries of key residues, which produce some favorable interactions with two inhibitors, are plotted in the complexes according to the lowest-energy structure from the MD trajectory. (**A**) pDI6W-MDM2 complex; (**B**) pDI6W-MDMX complex; (**C**) WK23-MDM2 complex; and (**D**) WK23-MDMX complex. The dashed line represents a hydrogen bond between the inhibitor and protein.

To gain a more-detailed insight into the effects of the specific active site residues on the inhibitor binding, structure and binding mode, analyses were carried out to complement the previous energy analyses. Hydrogen bond analyses based on MD simulations were also performed and the information was listed in Table 2. The decomposition analysis generates an inhibitor-protein interaction spectrum showing the interactions of the inhibitors with individual residues (Figure 6). Figure 7 depicts the relative positions of the inhibitors and correlated residues in the binding complex by using the lowest energy structure taken from the MD trajectories.

According to Figure 6A, eleven residues are involved in the main binding attractions, with the inhibitor-residue interaction stronger than 1 kcal·mol^−1^ for the MDM2-pDI6W complex. Structurally, the phenol of the residue Tyr67 and the phenyl of Phe19′ generate an almost paralleled π–π interaction (Figure 7A) and the distances of carbon atoms between two rings range from 3.65 to 6.79 Å [52], which produces an interaction energy of −2.07 kcal·mol^−1^ between Phe19′ and Tyr67. The alkyls of Ile61 and Met62 form many CH–π contacts with the phenyl of Phe19′ [53,54], which respectively corresponds to two interaction energies of −1.93 and −1.42 kcal·mol^−1^ of pDI6W with Ile61 and Met62. In addition, the nitrogen atom N in the backbone of Phe19′ provides a hydrogen atom H to construct a hydrogen bond with OE1 of Gln72 (Figure 7A and Table 2), which leads to a weak favorable binding energy of −0.88 kcal·mol^−1^. The occupancy of 71.34% of this hydrogen bond shows that it is stable during the simulation. Thus, four residues Tyr67, Met62, Ile61 and Gln72 build a hydrophobic pocket that matches the hydrophobic phenyl of Phe19′ to form a shape complementarity. The interaction energy of pDI6W with Val93 is −3.68 kcal·mol^−1^ and is the strongest among all residues, and this favorable energy may partly come from the CH–π contacts between the indole of Trp23′ and the alkyl of Val93 and partly from the CH–CH contacts [53,54] between the alkyl of Leu26′ and Val93. Except for the CH–π interactions from the indole of Trp23′ with the alkyl of Leu54 and the CH group of Gly58, the atom NE1 of Trp23′ forms one hydrogen bond with the backbone carbonyl oxygen of Leu54 (Figure 7A), the distance between the corresponding oxygen and nitrogen atoms is 2.91 Å and the occupancy is 97.20% (Table 2), which shows that Trp23′ produces the interaction energies of −3.27 and −1.3 kcal·mol^−1^ with the two residues Leu54 and Gly58, respectively. Therefore, Leu54, Gly58 and Val93 form the second hydrophobic pocket to which Trp23′ binds. The binding energies of pDI6W to His96 and Ile99 are −1.73 and −1.29 kcal·mol^−1^, respectively. This result is in agreement with the hydrophobic CH–π interaction between the alkyl of Leu26′ and the imidazole of His96 and the hydrophobic CH–CH contacts between two alkyls of Leu26′ and Ile99 (Figure 7A). Thus, four residues Leu54, Val93, His96 and Ile99 encircle the third hydrophobic pocket that accommodates the hydrophobic side chain of Leu26′. In addition, the imidazole of His73 and the indole of Trp22′ form a hydrophobic π–π interaction of −2.06 kcal·mol^−1^, and a strong hydrophobic CH–CH interaction of −3.11 kcal·mol^−1^ also exists between Thr27′ and Lys51 (Figure 6A and Figure 7A).

For the WK23-MDM2 complex, the number of the residues involving the Wk23-MDM2 interaction is less than the pDI6W-MDM2 complex. According to Figure 6C and Figure 7C, WK23 loses the hydrogen bond between Phe19′ and Gln72 and the hydrophobic interactions of pDI6W with the residues Thr27′ and Trp22′. The rest analysis is similar to the pDI6W-MDM2 complex. As seen from Figure 7A,C, the orientation of the residue Tyr100 points outward and the third hydrophobic pocket can be well formed to accommodate the side chain of Leu26′ and the ring R4 of WK23.

In the pDI6W-MDMX binding complex (Figure 6B and Figure 7B), the residues Ile60, Met53, Tyr66 and Gln71 shape the first hydrophobic pocket that matches the hydrophobic phenyl of Phe19′. Similar to the pDI6W-MDM2 binding, the pDI6W-Tyr66 binding energy is −2.25 kcal·mol^−1^, which structurally agrees with the strong π–π interaction between the phenyl of Phe19′ and the phenol of Tyr66. The hydrogen bond between the atom N of Phe19′ and the atom OE1 of Gln71 also contributes a weak favorable energy (Figure 7B and Table 2). The CH–π contacts between the alkyls of Ile60 and Met63 and the phenyl of Phe19′ may result in the energy contributions of −1.52 and −2.05 kcal·mol^−1^, respectively. The alkyl of Met53 not only produces many CH–π contacts with the indole of Trp23′ and CH–CH interactions with the alkyl of Leu26′, but also the atom O of Met53 forms a hydrogen bond with the atom NE1 of Trp23′ with the occupancy of 98.76%, which results in the pDI6W-Met53 binding energy of −2.92 kcal·mol^−1^ (Figure 6B, Figure 7B and Table 2). The indole of Trp23′ contacts the imidazole of His54 to generate the hydrophobic and aromatic stacking interaction of −1.59 kcal·mol^−1^, and also is close to the CH group of Gly57 to form the CH–π interaction of −1.52 kcal·mol^−1^ (Figure 6B and Figure 7B). In addition, the pDI6W-Val92 interaction energy is −2.31 kcal·mol^−1^, which may mainly come from the CH–π interaction with Trp23′ and the CH–CH contacts with Leu26′. Thus, Met53, His54, Gly57 and Val92 build the second hydrophobic pocket that accommodates the indole of Trp23′. According to Figure 6B, the interaction energy of pDI6W with Leu98 is −0.73 kcal·mol^−1^, which structurally corresponds to a number of CH–CH contacts between the alkyls of Leu26′ and Leu98 (Figure 7B). The aromatic ring of Pro95 is near the alkyl of Leu26′, which may be the main source of the pDI6W-Pro95 interaction (−1.31 kcal·mol^−1^). Thus, the residues Met53, Val92, Pro95 and Leu98 form the third hydrophobic pocket that matches the hydrophobic alkyl of Leu26′. As seen from Figure 7B, the residue Tyr99 points toward Leu26′ and prevent the alkyl of Leu26′ from moving into the deep of the pocket. Just for this reason, the interaction energies of pDI6W with the three residues Val92, Pro95 and Leu98 in MDMX are 2.37, 0.62 and 0.56 kcal·mol^−1^ weaker than one of pDI6W with Val93, His96 and Ile99 in MDM2, respectively. This result shows that Tyr99 certainly forms a steric clash with Leu26′ and produces an important effect on the inhibitor-protein binding, which has been proven by the previous analyses of binding free energies. In addition, the strong CH–CH contacts between Lys50 and Thr27′ also provide favorable contribution to the pDI6W-MDMX binding.

For the WK23-MDMX complex, the binding mode prediction of WK23 to MDMX is similar to the pDI6W-MDMX complex. It is observed that the number of the residues involving the WK23-MDMX interaction is less than the pDI6W-MDMX complex. By comparing Figure 6D with Figure 6B, WK23 loses the hydrogen bond between Phe19′ and Gln71 and the WK23-Lys51 interaction. Because the residue Tyr99 orients toward the ring R4 of WK23, Tyr99 prevents the ring R4 of WK23 into the deep of the hydrophobic pocket shaped by Met53, Val92, Pro95 and Leu98, Which is the main reason that the interaction energy of WK23 with Pro95 and Leu98 in MDMX is 0.87 and 0.42 kcal·mol^−1^ weaker than one of WK23 with His96 and Ile99 in MDM2, despite the binding energy of WK23 with Val92 of MDMX slightly higher than one of WK23 with Val93 of MDM2. This result shows that the orientation conflict of Tyr99 with the ring R4 of WK23 can produce important influence on the inhibitor binding. This work basically agrees with the previous studies [6,26,27,31].

Based on the above analysis, three important conclusions can be obtained: (1) the peptide inhibitors can generate more interaction contacts with MDM2/MDMX than the non-peptide inhibitors; (2) The steric clash formed by Tyr99 leads to the decrease in the inhibitor-MDMX binding affinity; (3) The π–π, CH–π and CH–CH interactions dominated by the shape complementarity drive the bindings of the inhibitors in the hydrophobic cleft of MDM2/MDMX.

### 2.5. Computational Alanine Scanning

**Figure 8:**
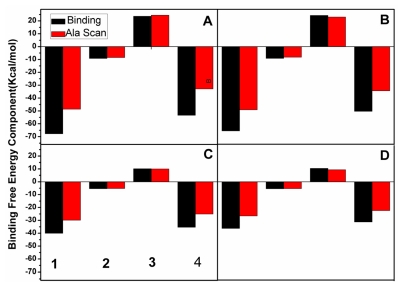
Changes of binding free energy components caused by alanine scanning. Binding of inhibitors to wild-type protein and mutated protein are represented by black and red, respectively. (**A**) pDI6W-MDM2 complex; (**B**) pDI6W-MDMX complex; (**C**) WK23-MDM2 complex; and (**D**) WK23-MDMX complex. Components are as follows: 1 van der Waals energy (Δ*E*_vdw_), 2 non-polar solvation energy (Δ*G*_nopol_), 3 polar interaction energy (Δ*G*_pol + ele_), 4 binding free energy (Δ*G*_gb_).

We performed alanine scanning on the inhibitor-MDM2/MDM2 complex to validate the contributions of free energy components to the binding. Because the polar solvation energy (Δ*G*_pol_) is closely related to the electrostatic term (Δ*G*_ele_), these two components were combined into a component: polar interaction component (Δ*G*_ele + pol_). Figure 8 shows the binding free energies and energy components of the complex with the mutated and wild protein, which includes van der Waals energy (Δ*G*_vdw_), non-polar solvation free energy (Δ*G*_nopol_), polar interaction component (Δ*G*_ele + pol_) and binding free energy (Δ*G*_gb_). The alanine scanning results in different effects on the separate energy component. As seen in Figure 8, the alanine scanning hardly influences the non-polar solvation energy component and polar interaction energy. However, it produces an obvious effect on van der Waals energy and the binding free energy. This is because the alanine scanning reduces the hydrophobic chain of the selected residues and causes the decrease in the number of van der Waals contacts between two inhibitors and MDM2/MDMX. Furthermore, Figure 8 suggests that the decrease in the binding free energy mainly comes from the decrease in van der Waals energy. Thus, it is concluded that van der Waals energy play an important role in the bindings of the inhibitors in the hydrophobic cleft of MDM2/MDMX, which is consistent with the previous free energy analyses.

**Figure 9:**
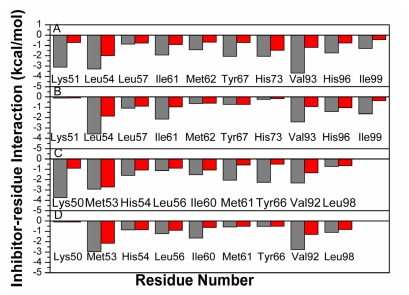
Changes of inhibitor-residue interaction energy caused by alanine scanning. Gray and red are used to represent the interactions of inhibitor with the residues of wild-type protein and mutated protein, respectively. (**A**) pDI6W-MDM2 complex; (**B**) WK23-MDM2 complex; (**C**) pDI6W-MDMX complex; and (**D**) WK23-MDMX complex.

To identify the binding hot spots of the protein and gain further insight into the contribution of each alanine mutation, we also calculated the change of the inhibitor-residue interaction caused by the alanine scanning (plotted in Figure 9). According to Figure 9, the alanine scanning leads to the decreases of more than 0.7 kcal·mol^−1^ in the interaction energies of the inhibitor with eight residues (Lys51, Leu54, Ile61, Met62, Tyr67, Val93, His96 and Ile99) for the pDI6W-MDM2 complex, four residues (Leu54, Ile61, Val93 and Ile99) for the WK23-MDM2 complex, six residues (Lys50, His54, Ile60, Met61, Tyr66 and Val92) for the pDI6W-MDM complex and four residues (Met53, Ile60, Val92 and Leu98) for the WK23-MDMX complex. This result is due to the decrease in the number of CH–π and CH–CH contacts or the loss of π–π interaction between the inhibitor and MDM2/MDMX caused by the alanine scanning. Although the oxygen atoms of Leu54 or Met53 form a hydrogen bond with the inhibitors, this oxygen atom belongs to the protein backbone. Thus, the alanine scanning does not influence this hydrogen bond. In a word, the π–π, CH–π and CH–CH interactions of the inhibitors with the proteins govern the bindings of the inhibitors in the hydrophobic cleft of MDM2/MDMX, which agrees with the previous analysis of binding modes.

## 3. Experimental Section

### 3.1. System Preparation

Initial structures of pDI6W-MDM2, pDI6W-MDMX and WK23-MDM2 complexes used in current computational studies came from X-ray structures (PDB entry: 3JZR, 3JZP, and 3LBK) in the protein data bank (PDB) [6,31]. The structure of WK23-MDMX complex was obtained by modifying the WK298-MDMX structure (PDB entry: 3LBJ) [6]. All missing hydrogen atoms of MDM2/MDMX and pDI6W were added by using the leap module in Amber10 software package [55]. All crystal water molecules in the PDB files were kept in the starting model. The force field ff99SB was applied to produce the force field parameters of the protein and crystal water molecules. The electrostatic potential of WK23 was calculated by using the Gaussian 98 package at the HF/6-31G* level [56]. Atom-centered partial charges were derived by using the RESP fitting technique in the AMBER [57]. The general AMBER force field (GAFF) [58] was used for the force field parameters of WK23, including the Lennard-Jones, torsion, bond angle terms. An appropriate number of chloride counterions were placed around the complex to neutralize the charges of the systems. Then, each system was embedded in a truncated octahedron box of TIP3P water molecules with a 10 Å buffer along each dimension [59]. To avoid edge effects, periodic boundary conditions were applied during the whole molecular dynamics (MD) simulation.

### 3.2. Molecular Dynamics Simulations

For each system, energy minimization and MD simulation were carried out using the sander module of the Amber 10 program. Before MD simulations, each system was subject to energy minimization in two stages to remove bad contacts between the complex and the solvent molecules. Firstly, the water molecules and counterions were minimized by freezing the solute using a harmonic constraint of a strength of 100 kcal·mol^−1^·Å^−2^. Secondly, each system was minimized without restriction. And each stage was performed using the steepest descent minimization of 1000 steps followed by a conjugate gradient minimization of 2000 steps. After the minimization, the system was then heated from 0 to 300 K in 100 ps and equilibrated at 300 K for another 100 ps. Finally, we run MD simulations on each system at 1 atm and 300 K for 9 ns to make sure that a stable trajectory for each of the simulated structures was obtained. During the simulation, the SHAKE method was applied to constraint the covalent bonds involving hydrogen atoms [60]. The Particle Mesh Ewald (PME) method was used for calculating the long-range electrostatic interactions [61,62]. The cutoff distances for the long-range electrostatic and van der Waals energy terms were set to 10.0 Å.

### 3.3. MM-GBSA Calculations

For each complex, a total number of 200 snapshots were taken from the last 2 ns of the MD trajectory with an interval 10 ps. The MM-PB/SA method and nmod module, which is implemented in Amber10, were applied to compute the binding free energies of the inhibitors to MDM2/MDMX. In this approach, the binding free energies (Δ*G*) are approximated by

(1) $$\Delta G=\Delta G_{\text{MM}}+\Delta G_{\text{sol}}-T\Delta S$$

where Δ*G*_MM_ is standard molecular mechanical energy in gas phase, Δ*G*_sol_ is the solvation free energy and *T*Δ*S* is a term involving the entropy effect. The molecular mechanical energy (Δ*G*_MM_) can further be expressed as

(2) $$\Delta G_{\text{MM}}=\Delta E_{\text{int}}+\Delta G_{\text{vdw}}+\Delta G_{\text{ele}}$$

where Δ*E*_int_, Δ*G*_vdw_ and Δ*G*_ele_ represent the internal energy contribution from bonds, angles and torsions, the van der Waals and electrostatic interactions in gas phase, respectively. The solvation free energy (Δ*G*_sol_) is further divided into two components:

(3) $$\Delta G_{\text{sol}}=\Delta G_{\text{pol}}+\Delta G_{\text{nopol}}$$

where Δ*G*_pol_ and Δ*G*_nopol_ are polar and non-polar contributions to the solvation free energy, respectively. The former component was computed using the modified GB model developed by Onufriev A *et al.* [48]. The dielectric constant inside the solute was set to 1.0 and 80.0 in the solvent in our calculations. Whereas the latter term was determined by

(4) $$\Delta G_{\text{nopol}}=\gamma \;\text{SASA}+\beta$$

where SASA is the solvent-accessible surface area and was calculated with the MSMS program [63]. In this work, the values for γ and β was set to 0.0072 kcal·mol^−1^·Å^−2^) and 0 kcal·mol^−1^, respectively.

The conformational entropies are important contributions to the inhibitor-receptor binding. Thus, normal-mode analysis was performed to compute the conformational entropy change upon the inhibitor binding. However, due to entropy calculations for large systems being extremely time consuming, we applied only 25 snapshots taken at an interval of 80 ps from the final 2 ns of the MD simulation to calculate the entropy contribution. Each snapshot was minimized with a distance-dependent dielectric function 4*R_ij_* (the distance between two atoms) until the root-mean-square of the energy gradient was lower than 10^−4^ kcal·mol^−1^·Å^−1^. The calculation error bars are the standard errors of the mean (SE)calculated using equation 5, in which STD is a standard deviation and *N* is the number of trajectory snapshots used in the calculation [64].

(5) $$\text{SE}=\frac{\text{STD}}{\sqrt{N}}$$

### 3.4. Inhibitor-Residue Interaction Decomposition

The inhibitor-residue interaction, which is valuable to qualitatively define the binding mechanisms of the four inhibitors to MDM2, was analyzed using a per-residue-based decomposition method [65] and approximated by:

(6) $$\Delta G_{\text{inhibitor}-\text{residue}}=\Delta G_{\text{vdw}}+\Delta G_{\text{ele}}+\Delta G_{\text{gb}}+\Delta G_{\text{surf}}$$

where Δ*G*_vdw_ and Δ*G*_ele_ are non-bonded van der Waals interactions and electrostatic interactions, respectively, between the inhibitor and each MDM2 residue in the gas phase and Δ*G*_gb_ and Δ*G*_surf_ are the polar and non-polar contributions to the inhibitor-residue interaction, respectively.

### 3.5. Computational Alanine Scanning Mutagensis

To determine the contribution of each residue in the interaction interface of the inhibitor-receptor binding and to study the detailed mechanisms at the energetic and atomic levels, computational alanine scanning was carried out on MDM2/MDMX, and the binding free energies of the inhibitors to the protein mutants were calculated by using the MM-GBSA method. The alanine mutant structures were obtained by altering the coordinates of the wild-type trajectory, which involves cutting atoms and truncating the mutated residue at *C*_γ_ by replacing with a hydrogen atom [66]. All parameters in the topology files of the mutated residues were accordingly replaced by the alanine residue parameters. The same 200 snapshots taken from the last 2 ns of MD trajectory with the time interval of 10 ps were used to calculate free energy. The key residues of the MDM2: Lys51, Leu54, Leu56, Ile61 Met62, Tyr67, Val93, His96 and Ile99, and the key residues of MDMX: Lys50, Met53, Ile60, Met61, Tyr66, Val92 and Leu98 were chosen for mutation. However, due to the significant difference in backbone conformations between proline and alanine, the Pro95 from the active site of MDMX was not selected [66].

## 4. Conclusions

In this work, MD simulation coupled with the MM-GBSA method has been carried out to probe the difference in the binding modes of the peptide and non-peptide inhibitors to MDM2/MDMX. The results confirm that the peptide inhibitors can produce more interaction contacts than the non-peptide inhibitors. The binding mode prediction of based-residue decomposition suggests that the π–π, CH–π and CH–CH interactions, dominated by the shape complementarity, govern the bindings of the inhibitors in the hydrophobic cleft of MDM2/MDMX. Lastly, we confirm that the existence of the potential steric clash formed by the residue Tyr99 due to energy and structure, make it possible to develop the potent dual inhibitors inhibiting the p53-MDM2/MDMX interaction.

## Figures and Tables

**Table 1:** Binding free energies computed by MM-GBSA method a.

Components b	pDI6W + MDM2		pDI6W + MDMX		WK23 + MDM2		WK23 + MDMX	

	mean	std c	Mean	std c	mean	std c	mean	std c
Δ*G*_ele_	−135.20	0.41	−144.89	0.32	−0.95	0.01	−1.78	0.05
Δ*G*_vdw_	−67.76	0.16	−65.49	0.17	−40.05	0.21	−36.26	0.31
Δ*G*_pol_	149.67	0.31	169.18	0.36	11.05	0.08	12.18	0.10
Δ*G*_nopol_	−9.18	0.05	−9.11	0.02	−5.36	0.21	−5.41	0.02
Δ*G*_ele + pol_	23.65	0.20	24.29	0.20	10.09	0.06	10.39	0.07
Δ*G*_gb_	−53.29	0.23	−50.31	0.24	−35.31	0.14	−31.28	0.14
−*T*Δ*S*	31.36	0.12	30.57	0.21	18.50	0.11	15.61	0.18
Δ*G*_bind_	−21.93		−19.74		−16.81		−14.89	
Δ*G*_exp_^d^	−10.5		−9.73		−8.26		−6.08	

a All values are given in kcal·mol^−1^;

b Component: Δ*G*_ele_: electrostatic energy in the gas phase; Δ*G*_vdw_: van der Waals energy; Δ*G*_nopol_: non-polar solvation energy; Δ*G*_pol_: polar salvation energy; Δ*G*_ele+pol_ = Δ*G*_ele_ + Δ*G*_pol_: polar interaction energy; Δ*G*_gb_ = Δ*G*_vdw_ + Δ*G*_nopol_ + Δ*G*_ele+pol_; −*T*Δ*S*: total entropy contribution; Δ*G*_bind_ = Δ*G*_gb_ − *T*Δ*S*;

c Standard errors of the mean;

d The experimental values Δ*G*_exp_ were derived from the experimental IC_50_ values in References [14,24] by using the equation Δ*G* ≈ −R*T*lnIC_50_.

**Table 2:** Hydrogen bonds formed between the inhibitors and MDM2/MDMX.

Protein	Donor a	Acceptor	Distance(Å) b	Angle(°) b	Occupancy(%) c
MDM2	Trp23′-NE1-HE1	Leu54-O	2.92	146.79	97.20
	Phe19′-N-H	Gln72-OE1	3.01	151.71	71.34
	WK23-N8-H75	Leu54-O	2.90	154.12	98.95
MDMX	Trp23′-NE1-HE1	Met53-O	2.89	140.29	98.76
	Phe19′-N-H	Gln71-OE1	3.01	150.94	69.66
	WK23-N8-H103	Met53-O	2.92	153.75	99.29

a The sign “′” represents the residue belonging to the peptide inhibitor pDI6W;

b The hydrogen bonds are determined by the acceptor···donor atom distance of less than 3.5 Å and acceptor···H-donor angle of greater than 120°;

c occupancy(%): to evaluate the stability and the strength of the hydrogen bond.
